# Comprehensive Analysis of the Implication of PGRMC1 in Triple-Negative Breast Cancer

**DOI:** 10.3389/fbioe.2021.714030

**Published:** 2021-10-22

**Authors:** Xin Xu, Xiangyan Ruan, Ying Zhang, Guiju Cai, Rui Ju, Yu Yang, Jiaojiao Cheng, Muqing Gu

**Affiliations:** Department of Gynecological Endocrinology, Beijing Obstetrics and Gynecology Hospital, Capital Medical University, Beijing Maternal and Child Health Care Hospital, Beijing, China

**Keywords:** PGRMC1, triple-negative breast cancer, mitochondrial function, TCGA, METABRIC

## Abstract

TNBC represents the most malignant subtype of breast cancer with heterogenicity and poor prognosis. PGRMC1 has been reported to predict worse prognosis and correlate with MHT mediated signal transduction in breast cancer, whereas its involvement in TNBC remains poorly explored. The purpose of the study was to explore the roles of PGRMC1 in TNBC. Bioinformatic approaches were performed to analyzed the expression of PGRMC1 among different subtypes of breast cancers using RNA-seq data from the TCGA, METABRIC and GEO databases. PGRMC1 mRNA expression and survival in breast cancer were analyzed. Furthermore, we analyzed the expression of PGRMC1 in TNBC by single cell RNA-seq data and immunohistochemistry. The expression of PGRMC1 in TNBC group was significantly higher compared with that of Luminal subtypes, especially in the epithelia cells, which was further proved by IHC at protein level. Better overall survival (*p* = 0.027) was observed in the patients with lower expression of PGRMC1. Different states of hormone and Her2 receptors contributed to the distinct functions of PGRMC1. In TNBC, PGRMC1 might play an important role in mitochondrial functions. In summary, this study revealed the correlation between PGRMC1 expression and its clinical significance in TNBC, probably through mitochondria-associated pathway, which may provide new ideas for prognosis and therapy of TNBC.

## Introduction

Breast cancer is the most common cancer (30% of all cancer cases) and the second leading cause of death among female cancers (15% of all cancer cases) in United States (Sun et al., 2019). Four subtypes of breast cancer have been classified as: luminal A [estrogen receptor (ER)+ and/or progesterone receptor (PR)+ and human epidermal growth factor receptor-2 (Her2)-], luminal B (ER+ and/or PR+ and Her2+), Her2-enriched (ER−, PR−, and Her2+) and basal-like triple-negative breast cancer. (ER−, PR−, Her2−) (Venkitaraman, 2010). TNBC accounts for 10–20% of breast cancer cases, and affects younger women with poor clinical prognosis (Chang-Qing et al., 2020). Nowadays, tumor resection, radiation therapy and chemotherapy are the main clinical options for TNBC treatment (Bergin and Loi, 2019). Recent advances with targeted therapies have achieved remarkable success in oncology. These include poly ADP ribose polymerase (PARP) inhibitors, which is used in patients with an inherited BRCA1 or BRCA2 mutation, and the programmed cell death 1(PD-1)/PD-1 ligand (PD-L1) checkpoint blockade (Garrido-Castro et al., 2019). Although treatment strategies have been significantly improved, prognosis of TNBC patients with metastasis remains disappointing, with a median overall survival (OS) about 13–18 months (Shah et al., 2012). TNBC is considered most aggressive and difficult to treat due to its heterogeneous in genomic, cellular, phenotypic aspects, and lacking of molecular targets, rapid metastasis (Wang et al., 2014; Cahill et al., 2016; Nik-Zainal et al., 2016; Karaayvaz et al., 2018; Wu et al., 2020; Núñez Abad et al., 2021).

PGRMC1 belongs to the membrane-associated progesterone receptor (MAPR) family. Roles of PGRMC1 have been well documented in non-genomic P4-responses in tissues of the female reproductive tract, regulation of cytochrome P450, steroidogenesis, vesicle trafficking, progesterone signaling, mitotic spindle and cell cycle regulation (Ahmed et al., 2012; Ruan et al., 2017; Cahill and Neubauer, 2021). Our team has been focusing on the association between PGRMC1 and breast cancer during the past decade. We found that PGRMC1 was overexpressed in human breast cancer. The overexpression was correlated with poor patient outcome (Ruan et al., 2019) and promotes the breast-cell proliferation upon combinational treatment of certain progestogens and estrogens (Clark et al., 2016; Li et al., 2019). Clark et al. demonstrated that PGRMC1 contributed to TNBC cell growth and survival *in vitro* and tumor development *in vivo* (Clark et al., 2016). Moreover, Diego A. Pedroza proved that PGRMC1 altered the PI3K/AKT/mTOR and EGFR signaling in TNBC cells, playing a crucial role in regulating the growth of cancer cells (Pedroza et al., 2020).

However, due to a relatively limit amount of research, the specific mechanism of PGRMC1 in TNBC is still un-known. In the present study, we employed bioinformatic analysis of the gene expression from TCGA-BRCA, METABRIC, GSE164458 and GSE118389 datasets to explore the roles of PGRMC1 in TNBC development.

## Materials and Methods

### Data Acquisition

Transcriptome and clinical data of breast cancer patients were downloaded from the TCGA database (https://tcga-data.nci.nih.gov/tcga/) and the METABRIC database (http://www.cbioportal.org/). The gene expression data of TNBC were downloaded from GEO, and there were one appropriate TNBC dataset from the GEO database (GSE164458) met the following criteria: 1) a total of more than 400 tumor samples; 2) annotated genes ac-counting for more than 90% of the total transcriptomes (*n* > 17,000); Details of the dataset were listed in Supplementary Table S1. Here, genes with adjusted p-value < 0.01 and fold change (FC) > 0.5 or < −0.5 were screened out. Genes of the intersection were considered as the DEGs. Single-cell transcriptome files of GSE118389 were downloaded to detect the expression of PGRMC1 in different cell clusters. Protein expression matrix of TCGA-BRCA was collected from CPTAC (https://proteomics.cancer.gov/programs/cptac).

### Differential Expression Analysis and Functional Enrichment Analysis

For the differential expression analysis, the DESeq2 algorithm was used to perform between PGRMC1-high and RPGRMC1-low TNBC samples (*p* < 0.05), |log2FC| > 0.5, and the difference of the mean value of normalized counts for each gene >500 to determine the significant difference. Upper quartile was considered as high expression and lower quartile were considered as low expression.

Gene Ontology (GO) biological process (BP), cellular component (CC), and molecular function (MF) enrichment analyses were performed. Gene Ontology (GO) enrichment analysis and Kyoto Encyclopedia of Genes and Genomes (KEGG) pathway analysis analyses of the DEGs were performed by DAVID. GO analysis included biological processes (BP), cellular component (CC), molecular function (MF). The KEGG database is a knowledge base for systematic analysis, annotation, and visualization of gene functions. GSEA was performed to elucidate key pathways involved in high vs. low PGRMC1 expression groups of basal breast cancer in the METABRIC dataset. GSVA (Gene Set Variation Analysis) (Hänzelmann et al., 2013) was used to calculate scores of gene sets that correlated with mitochondrial function (Rody et al., 2009).

### Survival Analysis

The association between OS and PGRMC1 expressed in breast cancer patients was determined using the online tool GEPIA2 (Tang et al., 2019).

### Single-Cell RNA-Seq Analysis

Single-cell trajectory reconstruction and Single-cell pseudotime trajectories were constructed with monocle. The “Seurat” package was used to perform the single-cell RNA-seq analysis (Butler et al., 2018). The batch effect from studies was removed with regularized negative binomial regression by the “Seurat” package. Dimension re-duction of the pre-processed matrix was performed by principal component analysis (PCA). Cluster biomarkers were found by the “Seurat” package. The tsne R package v0.1-3 was used for dimensionality reduction by t-distributed stochastic neighbor embedding (t-SNE). Single-cell trajectory reconstruction and Single-cell pseudo-time trajectories were constructed with monocle.

### Immunohistochemistry

49 TNBC tissue samples were obtained from Beijing Obstetrics and Gynecology Hospital, Capital Medical University. This study was approved by the Ethical commitee of the Capital Medical University, Beijing, China, with the trial number 2016-KY-080-01. The diagnosis was based on clinical and pathologic criteria. Immunohistochemical staining was performed to assess the expression of PGRMC1. Immunohistochemical protocols were carried out as previously described (Ruan et al., 2017; Cai et al., 2020a). IHC staining was semiquantitatively scored based on the percentage and intensity. The percentages of PGRMC1–positive cells were scored into five categories: 0, 0% of positive cells; 1, 1–10% of positive cells; 2, 11–33% of positive cells; 3, 34–66% of positive cells; and 4, 67–100% of positive cells. The positive reaction of PGRMC1 was graded from 1 to 3, where 1 indicates weak staining, 2 moderate staining, and 3 strong staining. The sum of the intensity and percentage scores was used as the final IHC score. For statistical analysis, the samples were grouped into negative (score <2) or positive (score ≥2). The IHC staining was analyzed by two observers blinded to clinical characteristics. The observer variation was less than 5%.

### Statistical Analysis

For comparisons, the Mann-Whitney test (between two groups) and the one-way analysis of variance (ANOVA) test (among multiple groups) were used for statistical significance estimated. For the correlation analysis between two continuous variables, the correlation coefficient (Spearman) was calculated to estimate the significance of the association. The prognostic *p* < 0.05 was considered to have the statistically significant difference. All the statistical calculations were performed using R software (https://www.r-project.org/) and the graphs display were performed by using GraphPad PRISM software (version 9.0, GraphPad Software, Inc.)

## Results

### The Expression and Prognostic Value of PGRMC1 in Breast Cancer

**FIGURE 1:**
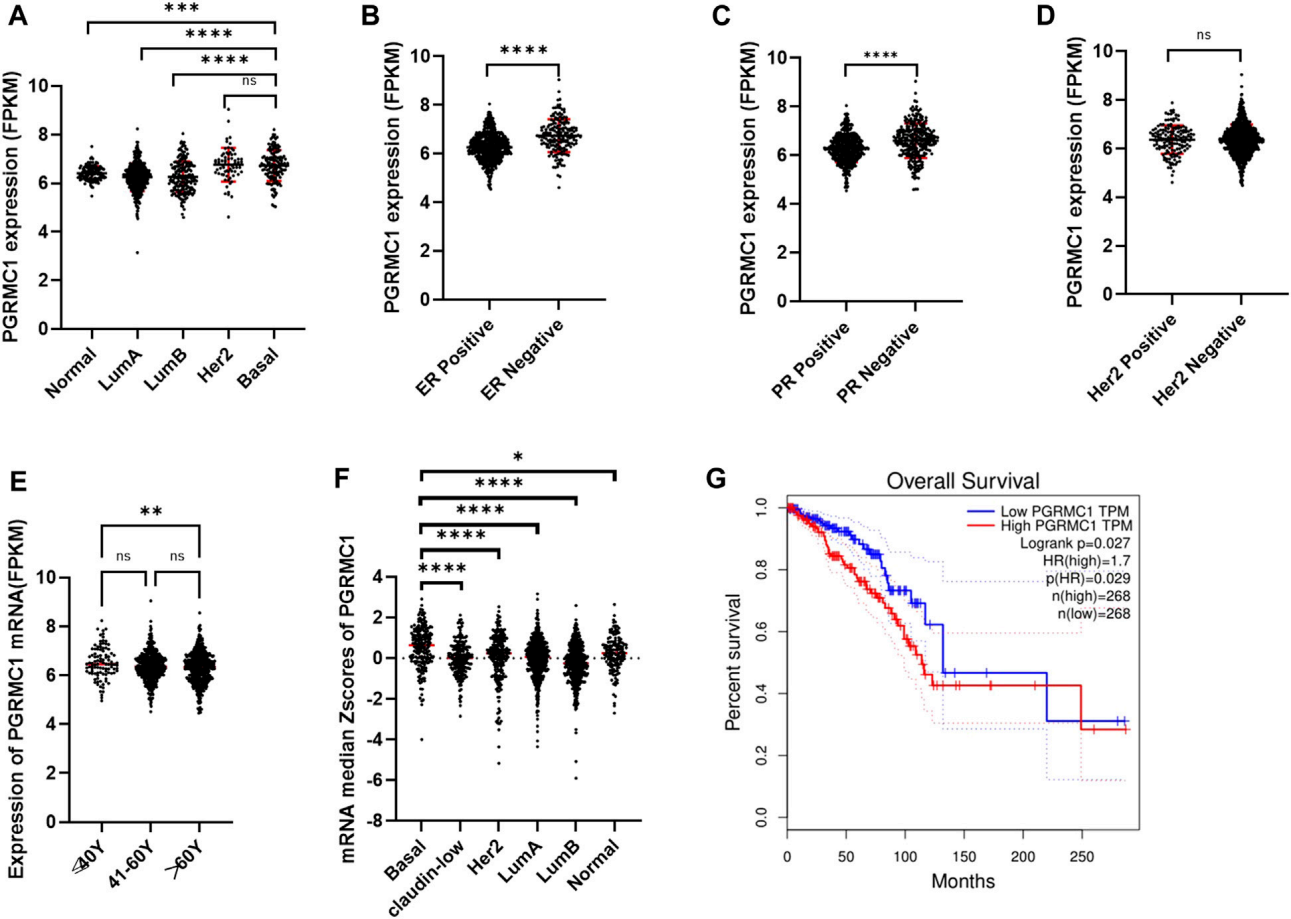
Expression characteristics and prognostic value of PGRMC1. **(A)** The mRNA expression of PGRMC1 in different types of breast cancer from TCGA-BRCA. **(B)** The mRNA expression of PGRMC1 in different ER states. **(C)** The mRNA expression of PGRMC1 in different PR states. **(D)** The mRNA expression of PGRMC1 in different Her2 states. **(E)** The mRNA expression of PGRMC1 in different groups of age. **(F)** The mRNA expression of PGRMC1 in different types of breast cancer from the METABRIC. **(G)** The survival curves for low and high PGRMC1 expressed groups by GEPIA2. (*means *p* < 0.01 ** means *p* < 0.001,*** means *p* < 0.0001,**** means *p* < 0.00001).

**FIGURE 2:**
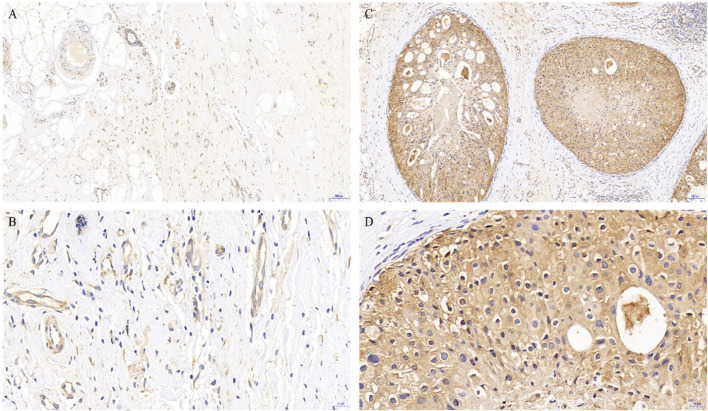
PGRMC1 expression in TNBC and paired normal breast tissues. Negative expression of PGRMC1 in normal breast tissues determined by immunohistochemistry (magnifications: A: 10×, B: 40×). Positive expression of PGRMC1 in breast cancer tissues determined by immunohistochemistry (magnifications: C: 10×, D: 40×). Signals for PGRMC1 have brown color; nuclei are blue. Dashed arrows represent the negative staining for PGRMC1.

To better understand the potential roles of PGRMC1 expression in breast cancer, we initially analyzed the ex-pression of PGRMC1 at transcript level in breast cancer patient cohort retrieved from the TCGA-BRCA and METABRIC dataset We found that PGRMC1 was associated with tumor subtypes, patient age, estrogen receptor (ER) status, progesterone receptor (PR) status and Her2 status. As shown in Figure 1, the mRNA expression of PGRMC1 in triple negative breast cancer group was significantly higher than that of LumA, LumB and the compared adjacent tissue (Figure 1A). the PGRMC1 expression was upregulated in the ER-negative group (ER−) (Figure 1B) and PR-negative group (PR−) (Figure 1C) in TCGA-BRCA. In the METABRIC dataset, the PGRMC1 was higher than that of other subtypes (Figure 1F). For survival analysis, GEPIA2 was used, which revealed that low expression of PGRMC1 in patients with breast cancer showed better overall survival (*p* = 0.027) (Figure 1G). Furthermore, the upregulated expression of PGRMC1 in breast cancer patients was also confirmed at protein level. In the CPTAC-TCGA-BRCA, the PGRMC1 expression was upregulated in the ER-negative group (ER−), PR-negative group (PR-) and Her2-positive group (Her2+) (Figure 1D). High PGRMC1 expression was observed in 35 of 49 TNBC patients (71.14%), which is slightly higher than that we have been reported in breast cancer of all subtypes (69.60%, 48/69) (Ruan et al., 2017) (Figure 2).

### Differential Expression Analysis and Functional Enrichment Analysis

**FIGURE 3:**
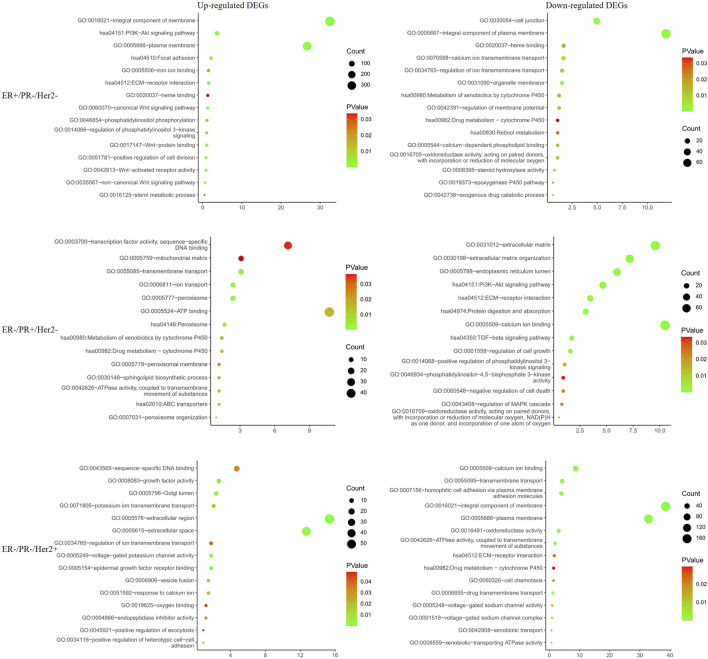
Functional enrichment analysis of the differentially expressed genes in different hormone and Her2 receptor status.

To further explore PGRMC1 related biological processes in different hormone and Her2 receptor status, we next performed the DEG analysis between PGRMC1 high- and low-expression samples which were single receptor positive based on TCGA-BRCA dataset. Samples (ER+/PR−/Her2−) were classified to high or low PGRMC1 levels by selecting the 25% top and bottom expressing tumors according normalized PGRMC1 expression values, respectively. Similarly, analysis (as described above) was performed within samples (ER−/PR+/Her2−) and samples (ER−/PR−/Her2+) (Supplementary Figure S1). Functional enrichment analysis was performed using DAVID database. We found that PGRMC1-related genes were mainly involved in PI3K-AKT signaling, transmembrane transport, drug metabolism, ATP binding related pathways (Figure 3). Interestingly, some pathways, like PI3K-AKT signaling pathway, enriched in up-regulated categories in ER+/PR-/Her2-samples. In contrast, it enriched in down-regulated categories in ER−/PR+/Her2-samples. It might be expected that PGRMC1 may play different roles in different hormone receptor states.

**FIGURE 4:**
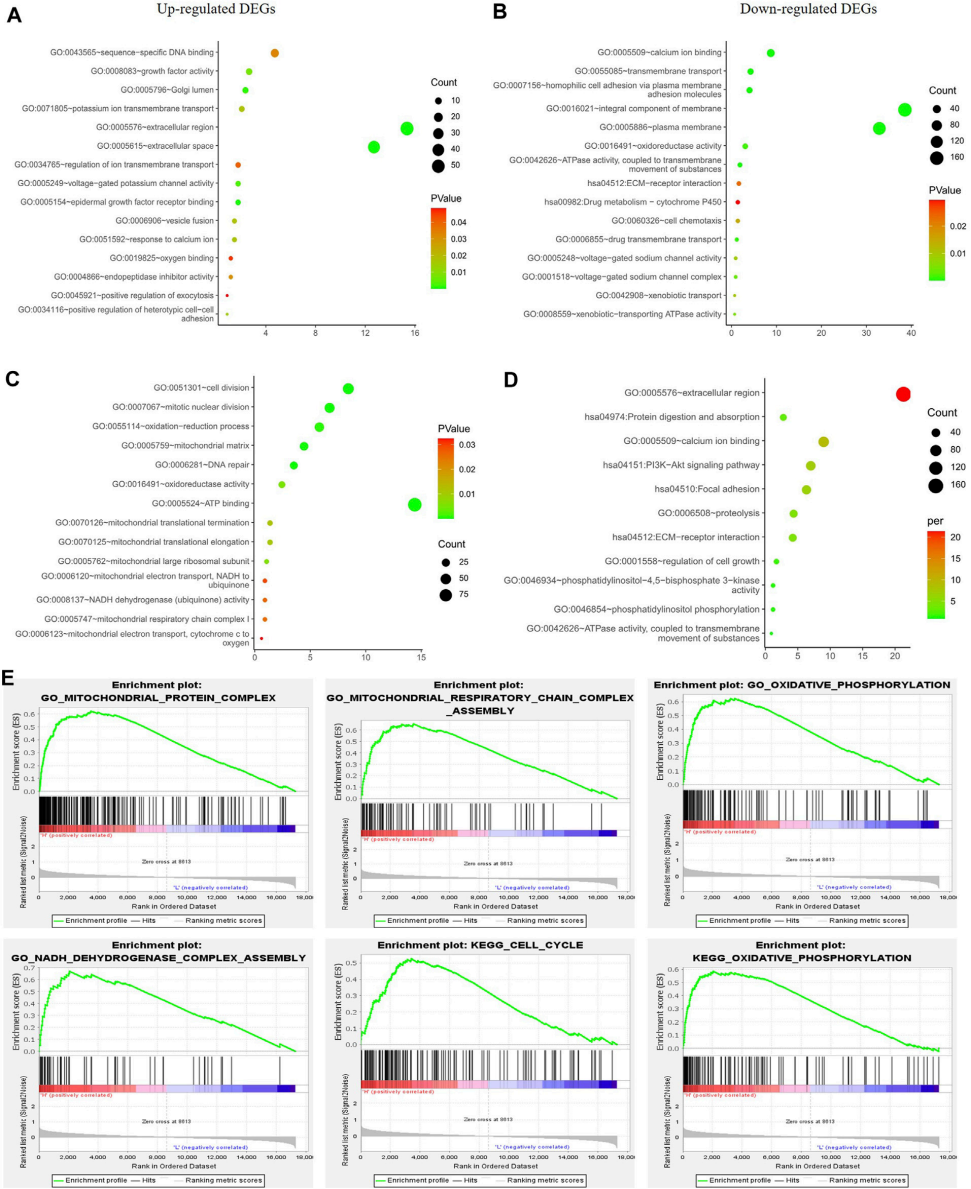
Function enrichment analyses for the differentially expressed genes between low- and high-PGRMC1 expression group in TNBC samples. **(A)** Function enrichment analyses of up-regulated DEGs in TNBC samples of TCGA-BRCA. **(B)** Function enrichment analyses of down-regulated DEGs in TNBC samples of TCGA-BRCA. **(C)** Function enrichment analyses of up-regulated DEGs in TNBC samples of GSE164458 dataset. **(D)** Function enrichment analyses of down-regulated DEGs in TNBC samples of GSE164458 dataset. **(E)** GSEA results for high PGRMC1 expression groups in TNBC samples of METABRIC dataset.

To exclude any indirect effects of hormone and Her2 receptor, we investigated the differential expression analysis and functional enrichment analysis in TNBC samples of TCGA-BRCA. The METABRIC and GSE164458 datasets were used to verify the performance of PGRMC1. Functional enrichment analysis showed that the overlapping DEGs were mainly enriched in mitochondrial functions, such as oxygen binding, mitotic nuclear division, mitochondrial respiratory chain complex (Figures 4A,C,E). PI3K-Akt signaling pathway enriched in down-regulated DGEs in GSE164458 dataset (Figure 4D), which was in contrast to the result of samples (ER+/PR-/Her2-) (Figure 3).

### Relationship Between PGRMC1 and Mitochondrial Functions

**FIGURE 5:**
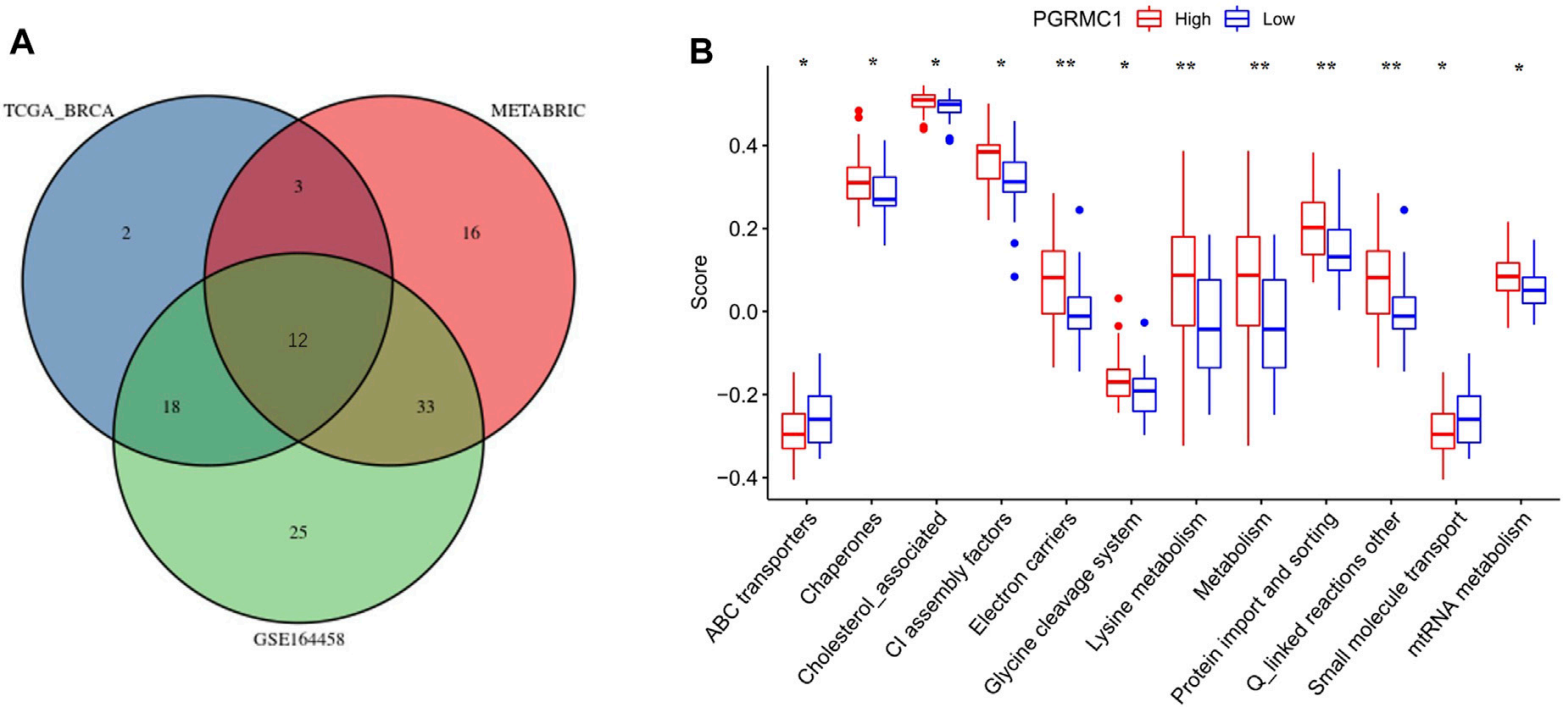
**(A)** Venn-diagram showing the result of GSVA analysis. **(B)** GSVA scores of the 12 mitochondrial pathways between high- (25%) and low-PGRMC1 (25%) expression samples in TNBC of TCGA-BRCA.

To further understand the role of PGRMC.1 in mediating mitochondrial functions, 149 mitochondrial pathways containing 1,136 human genes were derived from MitoCarta3.0 and defined as metagenes using the GSVA algorithm, implicating different types of mitochondrial functions in TNBC samples. Venn diagrams showed that 12 mitochondrial pathways were shared among the three datasets (Figure 5A). We found that PGRMC1 was positively correlated with Chaperones, Cholesterol associated, CI assembly factors, Electron carriers, Glycerol phosphate shuttle, Lysine metabolism, Metabolism, mtRNA metabolism, Protein import and sorting, as well as Q-linked reactions (Figure 5B). In summary, these findings indicated that PGRMC1 has important mitochondrial functions in TNBC.

### The Single-Cell Atlas of PGRMC1 Expression in TNBC Tissue

**FIGURE 6:**
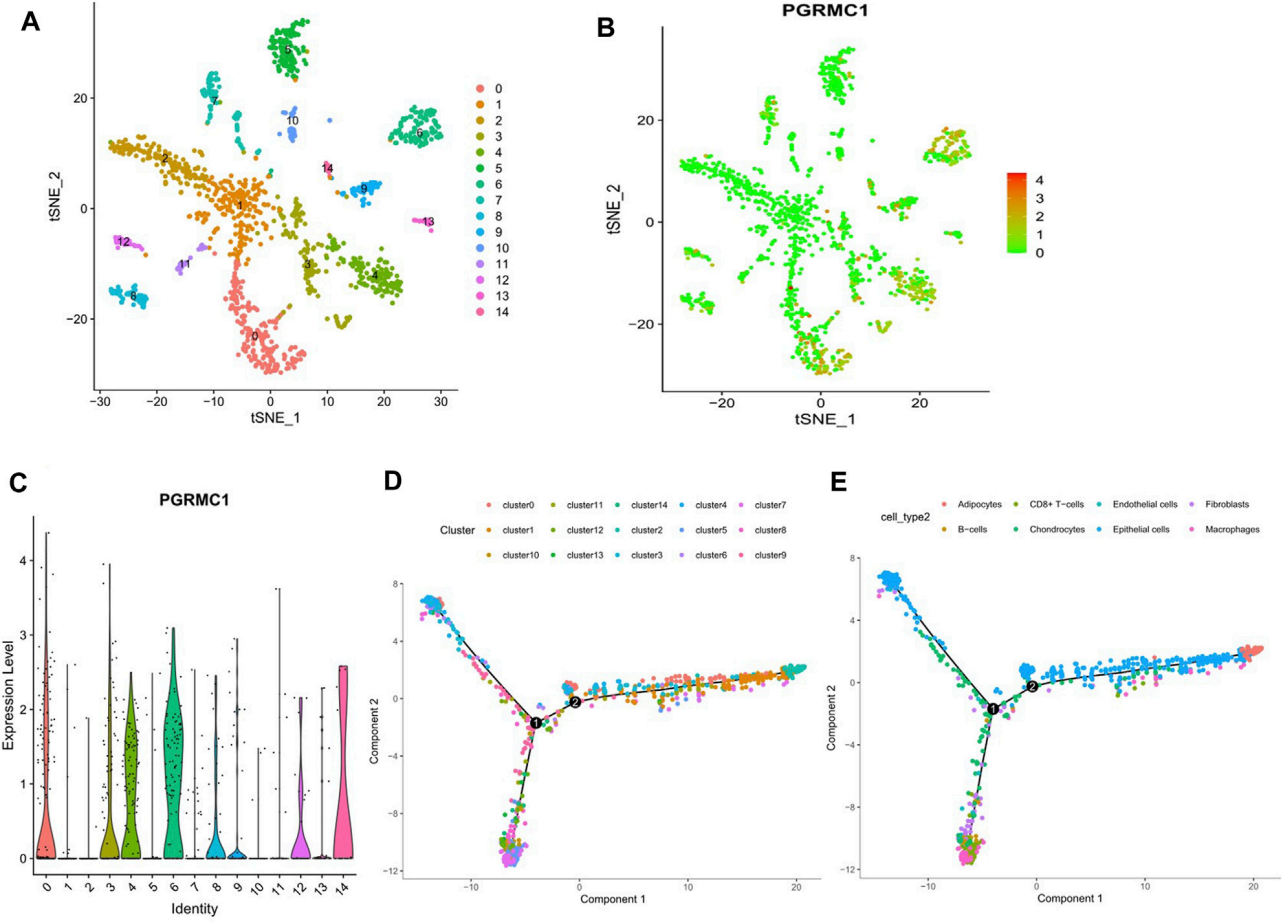
Results of reanalyzed Single-cell RNA sequencing. **(A)** Single-cell RNA sequencing identifies 15 clusters of cells in TNBC tissues. **(B, C)** Expression of PGRMC1 mRNA in different clusters. **(D)** Single-cell trajectory analysis reveals cell developmental pathways. **(E)** Relevant annotation tracks.

To determine the expression pattern of PGRMC1 in TNBC tissue, single cell RNA sequencing data of 1,534 cells in six fresh TNBC tumors from GSE118389 were re-analyzed. Clustering of gene expression profiles identified 15 sub-groups of malignant cells shared by multiple tumors (Figure 6A), and PGRMC1 was one of the upregulated cluster-specific marker genes in cluster 1 and 6 (Figure 6B). The majority of cells associated with cluster 1 and 6 were annotated as epithelia cells. Collectively, PGRMC1 was expressed in epithelia cell of TNBC.

## Discussion

The aberrant expression of PGRMC1 at both transcript and protein levels have been reported in multiple types of cancer, including breast (Neubauer et al., 2013; Li et al., 2019), ovarian (Peluso et al., 2008; Mir et al., 2012), as well as head and neck cancer (Zhao and Ruan, 2019). PGRMC1 may regulate the proliferation and progression of breast cancer cells, potentially by altering lipid metabolism and by activating key oncogenic signaling pathways, such as ERα expression and activation, as well as EGFR signaling (Asperger et al., 2020). In TNBC cells, PGRMC1 plays a prominent role in regulating the growth of cancer cells by altering the PI3K/AKT/mTOR and EGFR signaling mechanisms (Pedroza et al., 2020). In our previous studies, PGRMC1 expression was found to correlate with larger tumor size and lymph node metastasis in primary ER-negative breast cancer tissues (Ruan et al., 2017). PGRMC1 expression levels in cancer tissue were significantly correlated with PGRMC1 in blood, PGRMC1 may be valuable as a new tumor marker and may be superior to known tumor markers (Cai et al., 2020b). Our current study suggested that, PGRMC1 was highly expressed in TNBC cases at both transcript and protein levels. According to the analysis of the TCGA and the METABRIC database, significant higher expression of PGRMC1 were detected in TNBC than Luminal subtypes. Furthermore, the PGRMC1 expression might be associated with age, hormone and Her2 receptors. Interestingly, the PGRMC1 mRNA expression is also positive correlated with tumor stemness in RNA level score (Supplementary Figure S1). However further experimental studies would be required in order to clarify the biological role of PGRMC1 in tumor stemness associated pathway, such as OCT3/SOX2/NANOG/KLF4 (Finicelli et al., 2014). Meanwhile, high expression of PGRMC1 is correlated with a worse prognosis, while it was not significant in TNBC. A possible ex-planation might be the sample size, and the PGRMC1 phosphorylation may be involved in the clinical differences that underpin breast tumors of differing ER status (Neubauer et al., 2008; Gu et al., 2018). From the results of single cell sequencing of TNBC tissues, we found that the expression of PGRMC1 mRNA was increased in the epithelia cells, which is consistent with previous publication by Globinna Kim, demonstrated that PGRMC1 played a role in mouse mammary gland development, independent of PR (Kim et al., 2020).

To further explore the potential mechanisms, gene functional enrichment analysis was performed. Many terms were found related to response to cell cycle, PI3K-Akt signaling pathway, cell division, P53 associated pathway (Supplementary Figures S2D,E), and mitochondrial function. Partially, these results accord with findings of other studies before. Interestingly, some pathways, like PI3K-Akt signaling pathway, enriched in up-regulated DEGs when ER was positive. But the result was in contrast in TNBC samples. Pedroza DA demonstrated that PGRMC1 plays a prominent role in regulating the growth of cancer cells by altering the PI3K/AKT/mTOR and EGFR signaling mechanisms in both ER-positive and TNBC cells (Pedroza et al., 2020). A possible explanation for this could be that activation of various other signaling pathways could be functional in the different hormone states. This deserves further study. As we all know, P53 is a crucial tumor suppressor and transcription. In TCGA-BRCA, the PGRMC1 mRNA expression level in TP53-Mutant was significantly higher than that of TP53-NonMutant group (Supplementary Figure S2A). But in TNBC from both TCGA (Supplementary Figure S2B) and METABRIC (Supplementary Figure S2C), the difference aren’t significant. We also found that P53 associated pathway was enriched in GSEA analysis of METABRIC. This finding is consistent with that of Ji Yea Kim who demonstrated PGRMC1 suppresses the p53 pathways to promote the self-renewal of human pluripotent stem cell (Kim et al., 2018). The author also mentioned the wnt/β-catenin pathways. However, in our study, wnt associated pathway was enriched in the up-regulated DEGs of PGRMC1 high expression patients with ER+/PR-/Her2-. The sample size of TNBC and the effect of ER might be the reason.

Michael A. Cahill previously reported that PGRMC1 phosphorylation affected the abundance of hundreds of cellular proteins that induced altered mitochondrial form and function (Thejer et al., 2020). Our data found that PGRMC1 was positively correlated with Chaperones, Cholesterol associated, CI assembly factors, Electron carriers, Glycerol phosphate shuttle, Lysine metabolism, Metabolism, mtRNA metabolism, Protein import and sorting, as well as Q-linked reactions in TNBC samples. These pathways involved in core mitochondrial functions such as protein processing and respiration. Using pulldown assays and mass spectrometry, the functional annotation analysis categorized these proteins mainly into endomembrane system and mitochondria cellular components, both related to adenosine triphosphate (ATP) generation and transport activity, protein biosynthesis and posttranslational processing, vesicle trafficking, and protection against oxidative stress activities in human endometrial stromal cells (Salsano et al., 2020). Recent studies have reiterated the importance of metabolic reprogramming in various cancers. TNBC has been reported to have a greater glycolytic phenotype and showed an increase in choline and glutamate and a decrease in glutamine levels compared to ER + tumors (Mohanti et al., 1996; Pelicano et al., 2014). Metabolic plasticity of TNBC might suggest multiple potential adjuvant therapeutic targets. However, the underlying mechanisms be-tween PGRMC1 and mitochondrial function require further study.

## Conclusion

Our data suggests that PGRMC1 may play an important role in TNBC, and may be associated with mitochondrial function. However, further studies should be warranted for the underlying mechanisms between PGRMC1 and mitochondria in TNBC development.

## Data Availability

The datasets presented in this study can be found in online repositories. The names of the repository/repositories and accession number(s) can be found in the article/Supplementary Material.
