# Supplementary figures and images for: Comprehensive Analysis of the Implication of PGRMC1 in Triple-Negative Breast Cancer
Source: Front Bioeng Biotechnol. 2021 Oct 22;9:714030. doi: 10.3389/fbioe.2021.714030 (PMC8569863; doi:10.3389/fbioe.2021.714030)

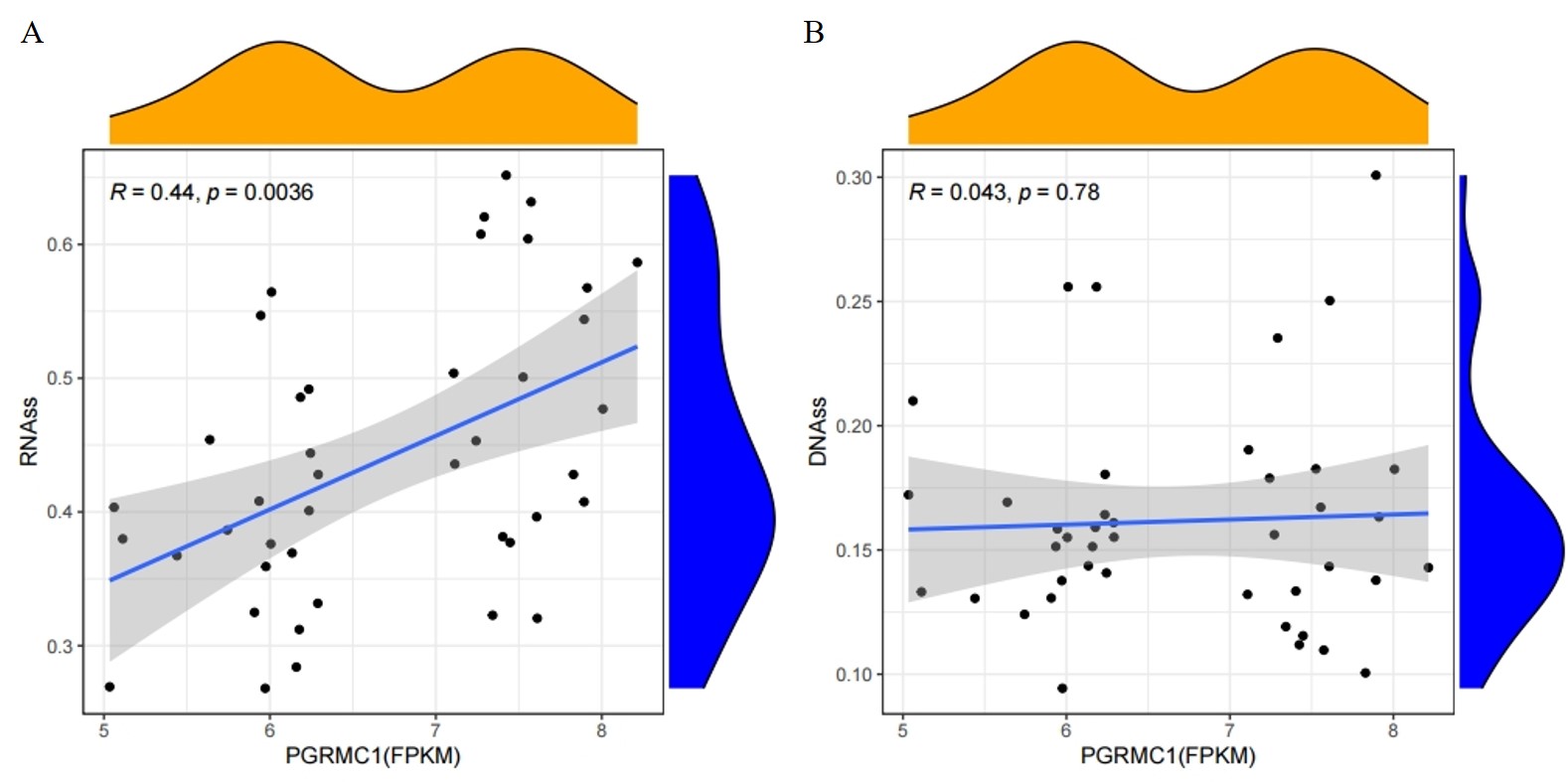

Supplement: Supplementary file 1 [file Image1.JPEG]

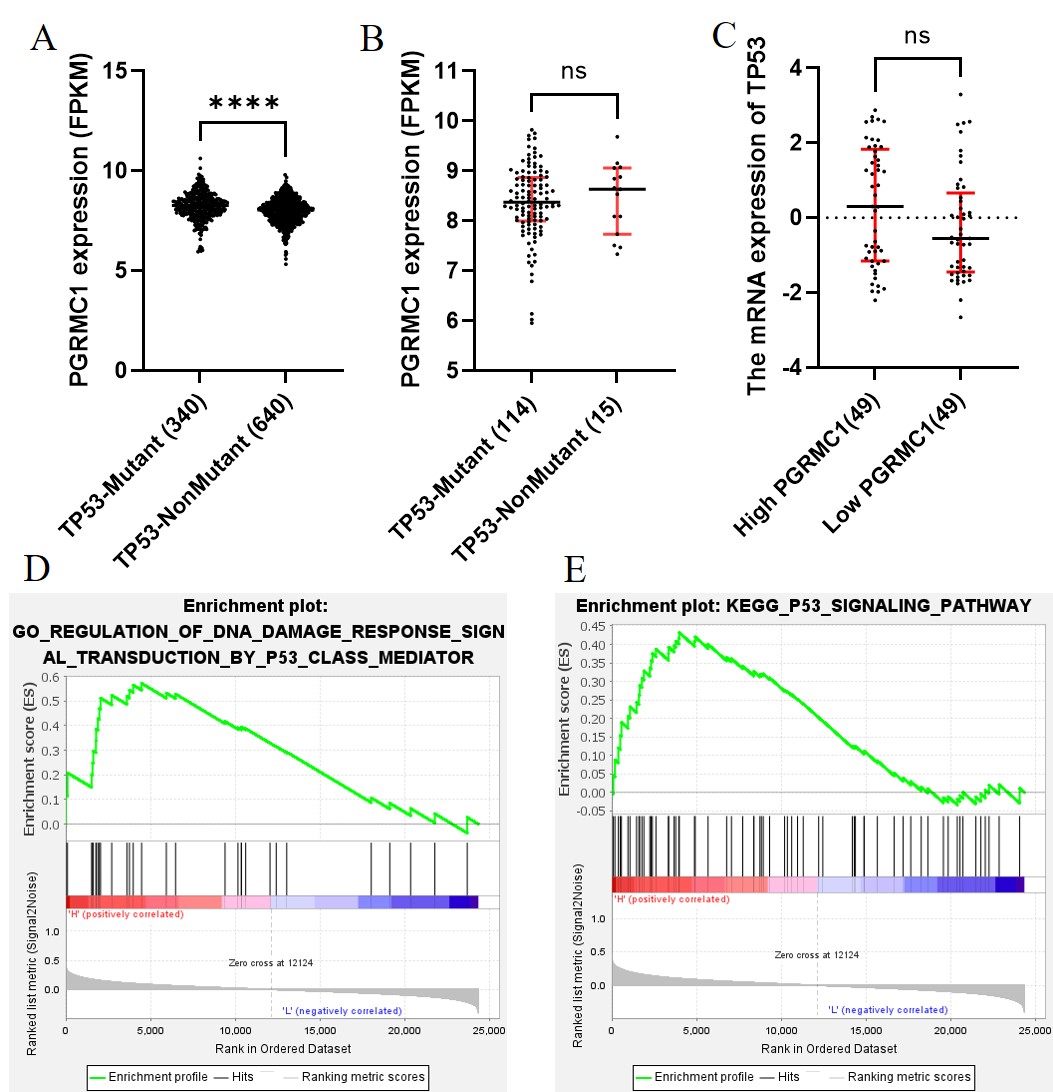

Supplement: Supplementary file 2 [file Image2.JPEG]
