# Supplementary material for: Comprehensive Analysis of the Implication of PGRMC1 in Triple-Negative Breast Cancer
Source: Front Bioeng Biotechnol. 2021 Oct 22;9:714030. doi: 10.3389/fbioe.2021.714030 (PMC8569863; doi:10.3389/fbioe.2021.714030)
Supplement: Supplementary file 3 [file DataSheet1.docx]

Supplementary Material

# Supplementary Tables

| Database | Source | Sample | Platform |
| --- | --- | --- | --- |
| GSE164458 | <https://www.ncbi.nlm.nih.gov/geo>  /query/acc.cgi?acc= GSE164458 | 482 | GPL21290 Illumina HiSeq 3000 (Homo sapiens) |
| GSE 118389 | <https://www.ncbi.nlm.nih.gov/geo>  /query/acc.cgi?acc= GSE62931 | 1534 | GPL9052 Illumina Genome Analyzer (Homo sapiens) |

**Supplementary Table 1.** The detailed information of the datasets.

| Clinicopathologic features | Amount |
| --- | --- |
| Age |  |
| ≤45 | 16 |
| 45~60 | 21 |
| >60 | 12 |
| Histological grade |  |
| Grade 1 | 9 |
| Grade 2 | 15 |
| Grade 3 | 25 |

**Supplementary Table 2.** Clinical parameters in 49 TNBC patients.

**Supplementary Figures**


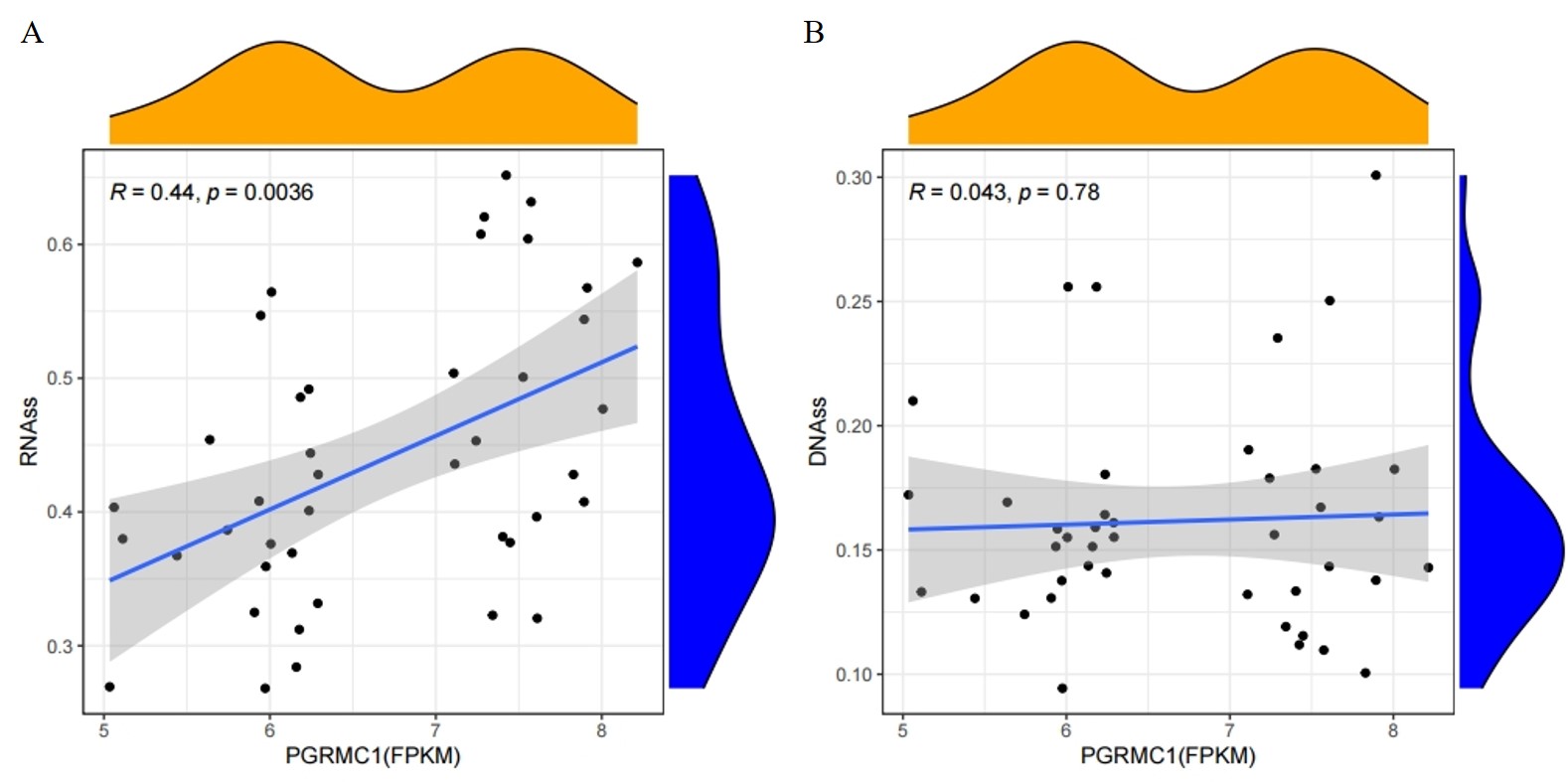
**Supplementary Figure 1**. The score of tumor stemness (RNAss) in TCGA-TNBC was significantly and positively correlated with PGRMC1 mRNA expression.


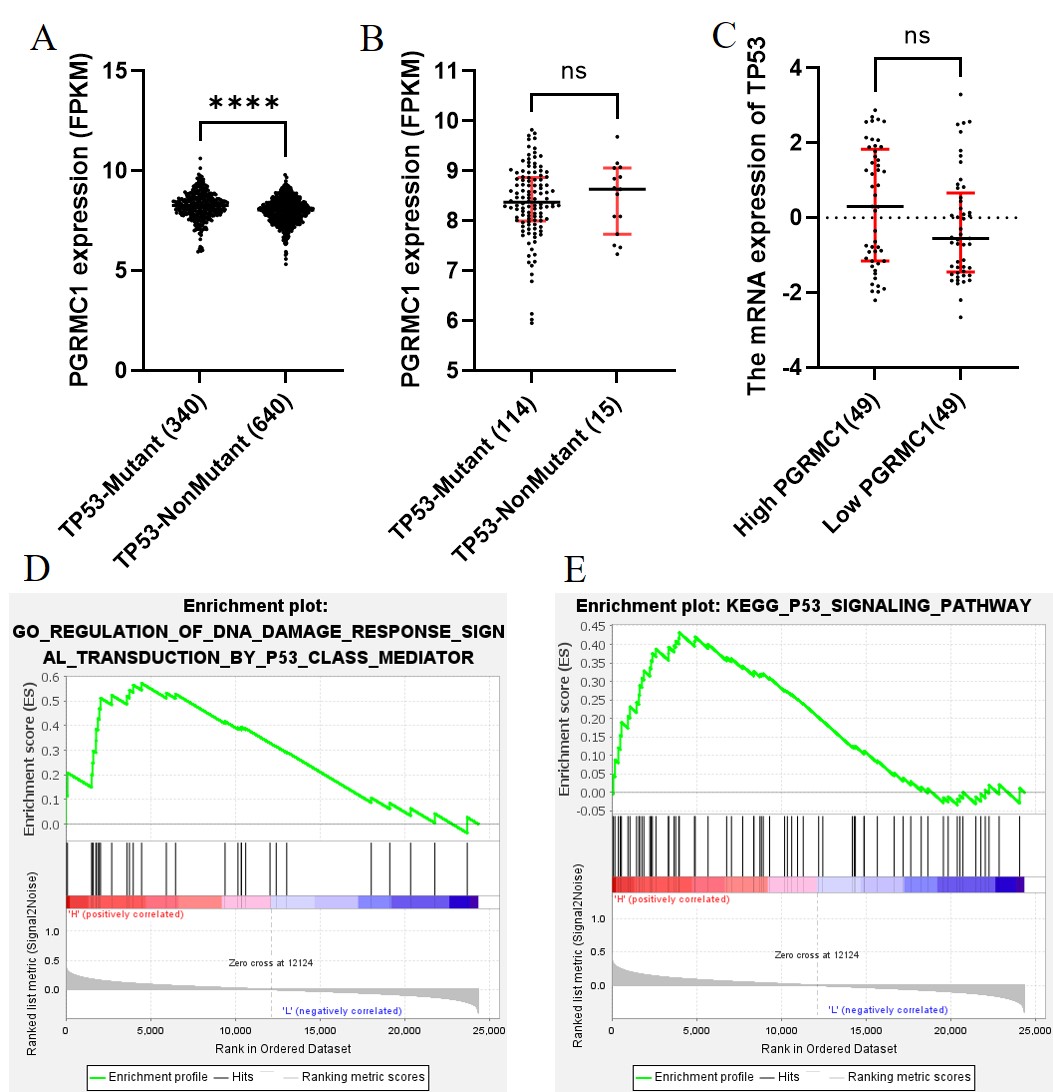


**Supplementary Figure 2.** The Correlation between PGRMC1 and P53 (gene symbol: TP53). (A) The PGRMC1 mRNA expression level in TP53-Mutant was significantly higher than that of TP53-NonMutant group in TCGA-BRCA. (**** means P < 0.00001) (B) No statistical difference is seen in PGRMC1 mRNA expression between TP53-Mutant and TP53-NonMutant group in TCGA-TNBC. (C) No statistical difference is seen in TP53 mRNA expression between High and low PGRMC1 expression group in METABRIC-TNBC. (D) GSEA results (GO) for high PGRMC1 expression groups in TNBC samples of METABRIC dataset. (E) GSEA results (KEGG) for high PGRMC1 expression groups in TNBC samples of METABRIC dataset.
